# Self-Rectifying Integrate-and-Fire Neuron and Collaborative Trim Training Framework for SNN-Based EEG Motor Imagery Classification

**DOI:** 10.3390/brainsci16060592

**Published:** 2026-05-30

**Authors:** Yifan Chen, Weihao Sun, Ming Meng

**Affiliations:** School of Automation, Hangzhou Dianzi University, Hangzhou 310018, China; 23070701@hdu.edu.cn (Y.C.); 232060320@hdu.edu.cn (W.S.)

**Keywords:** electroencephalography, brain–computer interface, spiking neural network, motor imagery

## Abstract

Background: Spiking neural networks (SNNs) have attracted significant attention in the field of brain–computer interfaces owing to their distinctive biological plausibility and energy efficiency advantages. However, the discrete nature of spikes renders gradient-based differentiation infeasible, making it difficult to directly obtain well-trained SNNs. A common approach is to transfer the weights from artificial neural networks (ANNs) to SNNs. However, this process introduces conversion errors that pose significant challenges. Methods: To address these challenges, we propose the self-rectifying integrate-and-fire (SRIF) neuron, which employs negative spikes to reduce asynchronism error and rectification spikes to diminish clipping error. Concomitantly, we propose a collaborative trim (CT) training framework that introduces a quantized network to perceive the weights and results of SNNs, which can further improve performance. Result: The proposed training methodology enables SNNs to achieve performance metrics comparable to those of ANNs in EEG-based motor imagery (MI) classification. Conclusions: Experimental results demonstrate that our method not only preserves the superior classification performance of ANNs but also leverages the superior energy efficiency and lower computational complexity of SNNs.

## 1. Introduction

Artificial neural networks (ANNs) exhibit superior performance in the domain of EEG signal decoding in a brain–computer interface (BCI) [1]. As deep learning advances, model performance improves but complexity increases, driving higher demands for memory and computation. Nevertheless, the human brain is remarkable for executing intricate perceptual and recognition tasks with tremendous energy efficiency, operating at approximately 20 watts [2]. Inspired by biological neurons, a new generation of spiking neural networks (SNNs) was proposed, which represents information using discrete spikes [3]. These discrete spike characteristics enable deep learning models to perform low-power and high-performance inference on specific neuromorphic platforms such as Intel Loihi [4], Truenorth [5], Spinnaker [6], etc. Nonetheless, training efficient SNNs remains challenging because spike non-differentiability prevents standard backpropagation.

Dan et al. [7] proposed a training method based on biological synaptic plasticity, spike timing dependent plasticity (STDP), to determine the connection strength of synaptic weights through the temporal differences in neuron firing. However, there is a problem of high computational complexity.

An alternative approach involves gradient replacement during backpropagation. For instance, Ding et al. [8] utilized the angular function to approximate gradients, enabling network weight training. This method can also achieve good performance on large-scale datasets, but it inevitably introduces errors in gradient calculation, leading to unstable convergence, and may also cause gradient vanishing or explosion in deep networks.

In comparison, implementing ANN to SNN conversion based on average spike firing rate encoding can avoid these problems. This strategy links activation values of ANNs with the average spike firing rate of SNNs, and converts pre-trained weights of ANNs into weights of SNNs, which can fully utilize the superior performance of ANNs and easily obtain SNNs with good performance. For example, Hu et al. [9] reduced conversion error in image classification by quantizing weights and introducing signed spiking neurons. It should be noted that this method imposes stringent requirements on conversion errors and results in SNNs that merely imitate ANNs without learning their inherent features.

Benefiting from SNNs’ strength in temporal signal feature extraction, researchers are paying growing attention to their application in BCI tasks. For example, Cappeci et al. [10] developed NeuCube, a universal architecture for brain data analysis, which has yielded effective results in various BCI tasks but still offers substantial room for performance enhancement. Liao et al. [11] proposed a lightweight and efficient neural network (LENet) for MI classification, which reduces the error during ANN to SNN conversion by minimizing the number of parameters and exhibits good performance. However, this strategy of avoiding conversion errors cannot directly and effectively solve the problem of conversion errors.

To address these issues, we design the self-rectifying integrate-and-fire (SRIF) neuron for ANN to SNN conversion and introduce a quantized intermediate network to reduce conversion errors. Meanwhile, we exploit the inherent feature extraction potential of SNNs through the collaborative trim (CT) training method, ultimately achieving high-performance and low-error conversion of SNNs for motor imagery (MI) classification tasks.

The main contributions of this study are as follows:

- We introduce the SRIF neuron capable of mitigating asynchronism and clipping error. We intuitively demonstrate the considerable drawbacks of data clipping error in a traditional IF neuron during ANN to SNN conversion. Our proposed SRIF neuron addresses these issues through secondary rectification, refining the spike firing behavior of the IF neuron and reducing the conversion error between the average spike firing rate of SNNs and the actual activation value of ANNs.
- We introduce a CT training method to prevent SNNs from merely imitating ANNs. This method uses a quantized network as an intermediate network to quantify the weights and activation values of ANNs, greatly reducing the difficulty of representing activation values of ANNs through the average spike firing rate of spiking neurons. At the same time, we expose the forward propagation process of SNNs to the quantized network, enabling it to perceive how the SNNs learn and adjust shared weights in a timely manner.
- We implement the conversion of common CNNs for MI classification into SNNs with lower error. By integrating SRIF and CT, we transformed EEGNet and ShallowConvNet used for EEG signal decoding into SNN form, and validated our method on BCI IV-2a and IV-2b. The results demonstrate that the SNNs obtained by our method feature low parameter counts, while achieving performance comparable to that of ANNs.

## 2. Related Works

### 2.1. Spiking Neurons

Spiking neurons are fundamental components of the SNNs. Their primary functions include integrating input currents, simulating biological neuron dynamics and resetting the membrane potential after firing. The most widely used spiking neurons in research include IF and LIF neurons. Among them, the equivalence of the IF neuron and ReLU activation function is often used in the ANN to SNN conversion [12]. This is a simplified model to simulate the activity of brain neurons. On the basis of IF, LIF has the mechanism of membrane potential leakage, which simulates membrane potential leakage in a resting state and is widely used in various SNNs [13].

An accurate information-representing spiking neuron is of vital importance. Stockl et al. [14] proposed few-spiking neurons to enhance information representation efficiency in IF neurons, enabling precise data encoding with reduced spike counts. However, this approach alters the data encoding method and is difficult to directly apply to the ANN to SNN conversion process based on average spike firing rate coding. Inspired by autapse, Wang et al. [15] introduced axon–dendrite and axon–soma circuits to dynamically constrain input currents and historical information, proposing the spatio-temporal circuit leaky integrate-and-fire (STC-LIF) model, which significantly improved the performance of SNNs in spatio-temporal prediction tasks. Nevertheless, this method only offers limited improvements in the ANN to SNN conversion process. Feng et al. [16] proposed a multi-level spiking neuron, using neurons with varying spike thresholds to form groups and create a multi-level firing mechanism, which enhances the expression capacity of neurons. Additionally, Guo et al. [17] introduced the ternary spiking neuron, enabling the spiking neuron to fire not only 0 and 1 spikes but also −1 spikes, thereby enhancing the information representation capability of neurons. Moreover, this study demonstrated that this ternary spiking neuron possesses the computational advantage of multiplication-free spike processing. In summary, these studies have provided ideas for solving the errors in the ANN to SNN conversion process by improving the information representation ability of spiking neurons. In this study, we focus on designing a spiking neuron with refined expression capabilities and effective compliance with the ANN to SNN training method by realizing more diverse spike firing patterns.

### 2.2. Training Approaches for SNNs

Current studies on SNN training primarily focus on two widely adopted methodologies: direct training using surrogate gradients and conversion from ANNs.

The surrogate gradient approach employs differentiable proxy functions to approximate the non-differentiable spike gradients during backpropagation. The selection of gradient approximation functions is critical, with common choices including the arctangent function, fast sigmoid function and exponential linear function.

To enhance surrogate gradient efficacy, Lian et al. [18] proposed an adaptive gradient surrogate range by leveraging the relationship between decay factors and membrane potential distributions, mitigating gradient vanishing and mismatch issues. Similarly, Ding et al. [19] proposed a variable time step backpropagation method, which divides training of SNNs into multiple stages. The time step size decreases progressively with each stage. After each stage, additional classifiers are added to reduce the loss of tag calculation, and alleviate the problems of mismatch between the proxy gradient and real gradient, ladder explosion and vanishing [15]. Nevertheless, challenges persist, including inaccurate gradient approximation, vanishing and exploding gradients, and high computational costs, particularly in complex networks or tasks.

Using well-trained ANNs to convert into SNNs is a fast and effective method to obtain SNNs. The ANN to SNN conversion paradigm, pioneered by Cao et al. [12], utilizes the functional equivalence between ReLU activations and the IF neuron. A key driving factor is the equivalence between average spike firing rates of SNNs and activation values of ANNs. However, discrepancies termed quantization error, clipping error and asynchronism error hinder effective conversion, especially in deep networks where such errors accumulate layer-wise, degrading SNN performance.

A considerable portion of the studies have focused on minimizing these errors. For instance, Yan et al. [20] proposed a clipped ReLU (CReLU) activation function to constrain activations of ANNs, albeit at the cost of ANNs’ performance. Hu et al. [9] introduced Fast-SNN, which employs additive power-of-two (APoT) quantization to discretize weights and activations of ANNs, enabling efficient two-stage conversion. Wang et al. [21] developed the DNISNM framework, incorporating an initialized spiking neuron and signed spiking neuron to address errors induced by spike discretization and asynchronicity. Despite these advances, converted SNNs largely mimic ANNs without fully leveraging SNNs’ specific advantages.

Among gradient surrogate training methods, numerous studies focus on developing efficient SNN training approaches, but they still cannot avoid the problems of the gradient agent itself, such as high training cost and low training efficiency. In the research on the transformation of ANNs to SNNs, although a well-trained SNN can be obtained through transformation at a low cost, there is still a problem that the advantages of SNNs cannot be brought into play. This is because when the ANN transfers the trained weights to the SNN, the SNN cannot perform any self-learning and only uses the trained weights to mimic the operation of the ANN. For these reasons, we introduce quantized network and CT training methods to build a low-error transformation method for the propagation process of the SNN to the quantized network, so that the quantized network can continuously adjust the shared weights during CT training, aiming to improve the final performance of the SNN.

### 2.3. Decoding EEG Signals with SNNs

The event-driven characteristics of SNNs have unique advantages for the feature extraction of sequential EEG signals, and they have therefore received attention from many researchers.

In current BCI implementations using SNNs, most studies use feature engineering to extract the features of EEG signals, obtain the feature maps, and then conduct spike coding using brain-derived data. Luo et al. [22] used three algorithms, discrete wavelet transform (DWT), variance, and fast Fourier transform (FFT), to extract EEG signals, and then analyzed and classified them by NeuCube architecture in spike form. For SNN-based EEG signal modeling, Zeng et al. [23] proposed a DNA-inspired double-helix hybrid symbiosis (DNA-HS) framework to realize bidirectional information interaction and collaborative optimization between ANNs and SNNs, which fully validates the effectiveness of SNNs in EEG signal modeling.

In addition, Liao et al. [11] proposed a lightweight and efficient LENet for MI classification, further proving the effectiveness of pure SNN architecture for EEG signal processing. In research on network architecture, Li et al. [24] proposed spike spatiotemporal neural architecture search (SSTNAS), which aims to search the SNN architecture suitable for each task for different tasks, so as to achieve effective analysis and processing of EEG signals. However, these methods inevitably raise model complexity alongside performance gains.

To sum up, in research on EEG signal processing using SNNs, most studies focus on how to partially use SNNs to improve the EEG decoding ability. In addition, numerous studies focus on training a SNN with excellent performance for EEG decoding, but the improvement of model complexity violates the lightweight nature of SNNs to some extent. Given the current state of the research, this study aims to develop a general-purpose methodology for converting well-trained ANNs in the BCI domain into lightweight, energy-efficient SNNs, thereby advancing the practical deployment of SNNs in BCI applications.

## 3. Methods

### 3.1. Self-Rectifying Integrate-and-Fire Neuron

#### 3.1.1. Error Analysis of IF Neuron Model

We use the IF neuron in the SNN, which serves as a model for converting the ReLU activation function in ANNs. The IF neuron is a classic spiking neuron, which imitates the behavior of biological brain neurons, and its core mechanism is to accumulate the membrane potential continuously. When the value of the membrane potential exceeds the threshold, it will fire a spike to the subsequent neurons. In the dynamic model of the IF neuron, the membrane potential before the firing of one spiking neuron of layer *l* at time step *t* is as follows:

(1) $$m_{l}\left(t\right)=v_{l}\left(t-1\right)+\sum_{i=1}^{n}w_{l,i}\cdot s_{l-1,i}\left(t\right)+b_{l},$$

where $v_{l}$ represents the membrane potential after the firing of this spiking neuron at time step t−1, $s_{l-1,i}$ represents the spike firing of neuron *i* in layer l−1, $w_{l,i}$ represents the weight of the connection between this neuron and neuron *i* in layer l−1 and $b_{l}$ represents the bias of this neuron.

Subsequently, the membrane potential $m_{l}$ is compared with the threshold $V_{th}$ to determine whether spike $s_{l}\left(t\right)$ is fired:

(2) $$s_{l}\left(t\right)=\left\{\begin{array}{cc} 1 & m_{l}\left(t\right)\geq V_{th},\\ 0 & m_{l}\left(t\right)<V_{th}. \end{array}\right.$$

In this study, we used soft reset instead of hard reset to calculate the membrane potential after the firing $v_{l}\left(t\right)$. Hard reset sets the membrane potential to a constant baseline, typically 0, while soft reset subtracts the threshold from the membrane potential when it exceeds the threshold. This mechanism has been shown to further preserve the information representation ability of spiking neurons [25]. The $v_{l}\left(t\right)$ is defined as follows:

(3) $$v_{l}\left(t\right)=m_{l}\left(t\right)-V_{th}\cdot s_{l}\left(t\right).$$

Given a spike train with *T* time steps, the average spike firing rate of spiking neuron is formulated as:

(4) $${\bar{s}}_{l}=\frac{\sum_{t=1}^{T}s_{l}\left(t\right)}{T}.$$

When using the IF neuron to express the activation value of ANN, the error is mainly embodied in clipping error and asynchronism error. Clipping error can be expressed in two forms. One is the average spike firing rate of SNN. Because its discrete form always has a certain degree of error when representing the activation value of ANN, this error can be optimized through the quantized network. Another form of error is that the actual activation value may exceed the maximum value of the average spike fire of the spiking neuron when the weight is shared. Through a simple example, we can intuitively understand the clipping error that the activation value is greater than the maximum spike firing rate. For example, for any layer, assume that the activation value *a* of ANN is 1.25, which is usually derived from the linear transformation of the Batch Normalization (BN) layer. When T=4, and the spike threshold $V_{th}$ is 1, the corresponding average spike firing rate has five discrete values: $\left\{0,0.25,0.5,0.75,1\right\}$. At this time, there is an error of at least 0.25 between the activation value and the average spike firing rate. It should be noted that in the error introduction here, the calculation of average spike firing rate does not involve the membrane potential accumulation and threshold judgment process of specific IF neuron, but only considers the error of the IF neuron in representing the activation value under the ideal state.

**Figure 1:**
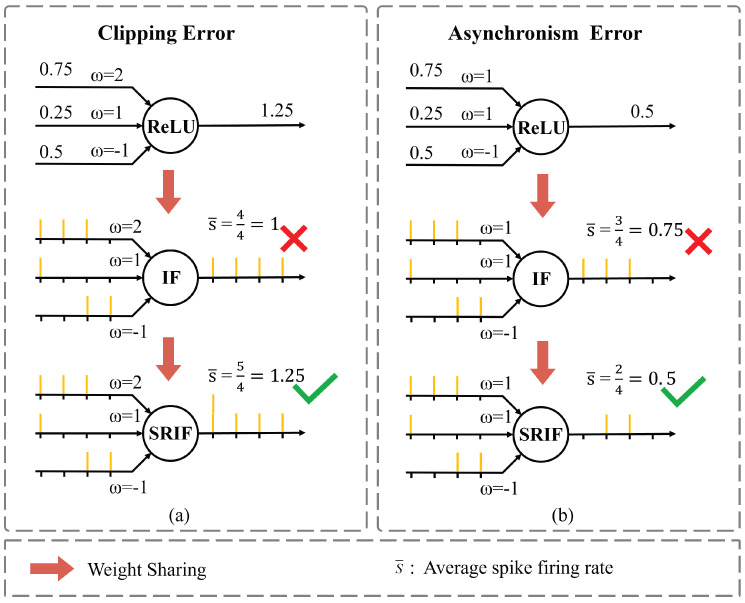
Clipping error and asynchronism error arise when IF neurons approximate ANN activation values via their average spike firing rate. (**a**) Residual membrane potential leads to a clipping error. (**b**) IF neurons fail under specific spike timing conditions. The SRIF model addresses these issues by correcting anomalous spikes through a secondary firing round.

This error can be visually represented by Figure 1a. In addition, in the ANNs to SNNs scenario, the asynchronism error is mainly reflected in the fact that the actual value of the average spike firing rate is greater than the theoretical value, which is mainly caused by the timing of the spike delivery. Figure 1b shows the source of this error. When the spikes that have a positive effect on the membrane potential of spiking neurons are concentrated in the previous time steps, the situation of low membrane potential caused by the spikes that have a negative effect on the membrane potential in the subsequent time steps may be ignored. Indeed, it is readily apparent that when *T* is made arbitrarily large, these errors exert a negligible influence on network performance; yet such a strategy directly undermines the low-cost premise that motivates the use of spiking neural networks.

#### 3.1.2. Self-Rectifying Integrate-and-Fire Spiking Neuron Model

To address clipping error and asynchronism error, we propose the SRIF neuron model, the core of which is that after the IF neurons fire out the spike, it adjusts the spike fire through rectification operation. For asynchronism error, we introduce a negative spike to reduce the average spike firing rate, and for clipping error, we propose spiking the neuron with a rectification spike. The purpose of the rectification spike is to reduce the clipping error and asynchronism error in the case of fewer time steps. Different from the idea of designing multiple thresholds for neurons, we retain the canonical integrate-and-fire (IF) model and seek to ensure that its native spiking behavior is accurately reproduced, as formalized in Equation (5). We calculate the residual membrane potential of the first stage of IF neuron spike for the rectification stage of spike calculation. The first stage spike calculation rules are defined as follows:

(5) $$\begin{array}{c} m_{l}^{f}\left(t\right)=v_{l}^{f}\left(t-1\right)+\sum_{i=1}^{n}w_{l,i}\cdot s_{l-1,i}^{f}\left(t\right)+b_{l},\\ v_{l}^{f}\left(t\right)=\left\{\begin{array}{c} \frac{V_{th}}{2}\;t=0,\\ m_{l}^{f}\left(t\right)-V_{th}\cdot s_{l}^{f}\left(t\right)\;t\neq 0, \end{array}\right.\\ s_{l}^{f}\left(t\right)=\left\{\begin{array}{cc} 1, & m_{l}^{f}\left(t\right)\geq V_{th},\\ 0, & m_{l}^{f}\left(t\right)<V_{th}. \end{array}\right. \end{array}$$

where $m_{l}^{f}$, $v_{l}^{f}$ and $s_{l}^{f}$ denote the membrane potential before the firing processing, membrane potential after the firing processing and spikes. Unlike traditional IF neuron, we initialize $v_{l}^{f}$ at half the threshold potential, which proved to be a simple method to reduce the clipping error [21].

The rectification stage rectifies suboptimal spike patterns generated in the initial stage. The calculation rule of membrane potential $m_{l}^{r}$ is similar to that of the first spike firing stage:

(6) $$m_{l}^{r}\left(t\right)=v_{l}^{r}\left(t-1\right),$$

where *r* represents the rectification stage. In this stage of spike calculation, the initial membrane potential is the postsynaptic potential at the last moment of the first stage. The calculation of membrane potential reset is as follows:

(7) $$v_{l}^{r}\left(t\right)=\left\{\begin{array}{c} v_{l}^{f}\left(T\right)\;t=0,\\ m_{l}^{f}\left(t\right)-V_{th}\cdot s_{l}^{f}\left(t\right)\;t\neq 0. \end{array}\right.$$

The operating mechanism of biological neurons indicates that asynchronism errors can be resolved through compensation of negative spikes. Izhikevich et al. [26] showed that thalamic cortical neurons have multiple emission thresholds, with higher thresholds around −60 mV and lower thresholds around −90 mV. When thalamic cortical neurons receive positive current like neuronal membrane potentials reaching higher thresholds, they will emit negative spikes for membrane potential compensation. This mechanism inspired us to design a historical membrane potential to track the spike firing at previous time steps, in order to correct asynchronism errors during the spike firing process. The historical membrane potential $h_{l}$ is:

(8) $$h_{l}\left(t\right)=\sum_{i=0}^{t-1}h_{l}\left(i\right)+s_{l}^{f}\left(t\right).$$

At t=0, $h_{l}\left(t\right)$ is initialized to zero. The final spike output is calibrated to minimize clipping error and asynchronism error. The specific spike firing calculation is as follows:

(9) $$s_{l}\left(t\right)=\left\{\begin{array}{c} s_{l}^{f}+1\;m_{l}^{r}\left(t\right)\geq V_{th},\\ s_{l}^{f}\;m_{l}^{r}\left(t\right)<V_{th},\\ s_{l}^{f}-1\;m_{l}^{r}\left(t\right)<-V_{th}\;\mathrm{and}\;h_{l}\left(t\right)>0. \end{array}\right.$$

**Figure 2:**
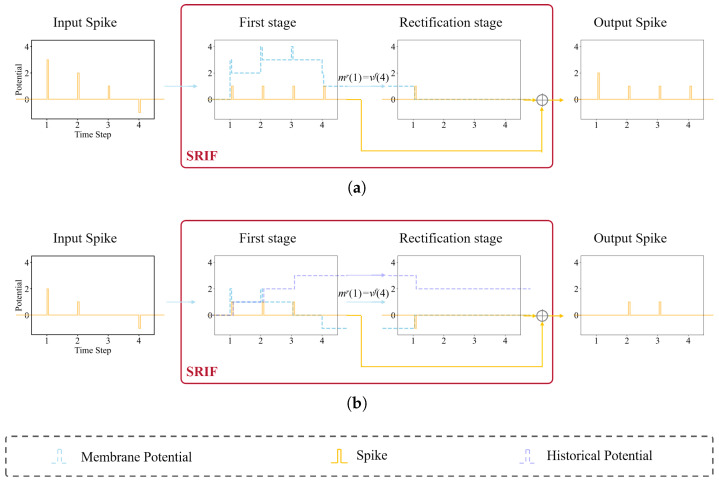
The calculation process of SRIF. (**a**) Eliminates clipping error. (**b**) Eliminates asynchronism error. A second spike firing round clears the residual membrane potential left by the first round, aligning the average firing rate of the SNN with the activation value of the ANN.

Compared with IF neurons, the advantages of SRIF are mainly reflected in two aspects. First, two stages of spike calculation can improve the fine representation ability of neurons under the same number of time steps. In addition, the advantage is that in the process of secondary rectification, the historical membrane potential information is used to rectify the spike firing to reduce the asynchronism error. Notably, our negative spike mechanism differs from the ternary spiking neuron proposed by Guo et al. [17]. The negative spike is not a mirror image of the positive spike, and does not play a role in the expression of key information. It only repairs the excessive membrane potential to avoid the actual average spike firing rate being greater than the ideal average spike firing rate. In actual operation, the expression of negative spike is limited, which is mainly caused by the average spike firing rate as the key conversion factor in the ANN to SNN conversion method. Because of this, we redesigned the firing rules for negative spikes. We still want to retain the equivalence of IF and ReLU, which is the basis of ANN to SNN conversion. Consequently, we augmented the firing criteria for negative spikes by incorporating additional constraints based on historical spike events, thereby preventing the average firing rate of the IF neuron from becoming negative. Ultimately, the operational process completed by SRIF will be fully reflected in Figure 2.

One of the important characteristics of EEG signal is its high time resolution. Therefore, it is an important step for the follow-up work to refine the representation of EEG in SNNs. The SRIF provides a computing margin in terms of refined representation to a certain extent, making the spiking neuron have a richer, more flexible and more detailed way to represent EEG signals.

### 3.2. Leverage the Advantages of SNN

#### 3.2.1. Quantized Network

Using a simple IF neuron to convert ANNs to SNNs has been popular in previous studies on SNNs. On this basis, it is still difficult to achieve low conversion error only by modifying the spiking neuron. This is because the continuity of the ANNs’ activation value makes the spiking neuron need a higher *T* to achieve a smaller error, and the higher *T* makes the computing advantage of SNNs no longer exist. We notice that several studies introduced quantitative ANN into the SNN training process, which can reduce the clipping error in the conversion process, and achieved excellent results [9,22].

The core concept of quantized networks is to discretize the weight and activation value of ANN by clipping. For example, the quantized *b*-bit unsigned integer has discrete values $\left\{0,1,\ldots ,2^{b-1}\right\}$. When $T=2^{b-1}$, the number of spikes $\left\{0,1,\ldots ,T\right\}$ and the quantized value are the smallest in clipping error [9]. In this study, we used uniform clipping to quantify the activation value. The calculation is defined as:

(10) $$Q_{l,i}=\frac{V_{th}}{T}\mathrm{clip}\left(\mathrm{round}\left(\frac{T\cdot x_{l,i}}{V_{th}}\right),0,T\right),$$

where $Q_{l,i}$ is the activation value of the neuron *i* in layer *l* after quantification, $x_{l,i}$ is the original activation value of the neuron *i* in layer *l*, round() is a rounding operation and clip(x,min,max) is a clipping operation that saturates *x* within range [min,max]. For the activation value, in order to reduce the clipping error as much as possible and make it convenient to use the average spike firing rate to represent the activation value, uniform clipping is an efficient and convenient operation, and this method has achieved remarkable results in the image domains [27]. In addition, we employ the additive power-of-two (APoT) clipping method proposed by Li et al. [28] to quantize the weight value, because APoT is more suitable for quantizing weights that follow bell-shaped and long-tailed distributions. The weight between the neuron *i* in layer l−1 and the neuron *j* in layer *l* is defined as:

(11) $$w_{l,(i,j)}=\sum_{k=0}^{K-1}\alpha_{k}\cdot 2^{k},$$

where *K* is the number of quantized digits. $\alpha_{k}$ is usually ±1 or another integer value, which is used to adjust the size of the power term of each two. Here, we do not introduce the details of the quantitative method, but simply introduce the core idea of the quantitative method and the advantages of using the quantitative network to transform ANNs to SNNs.

#### 3.2.2. Cooperative Trim Training Framework

**Figure 3:**
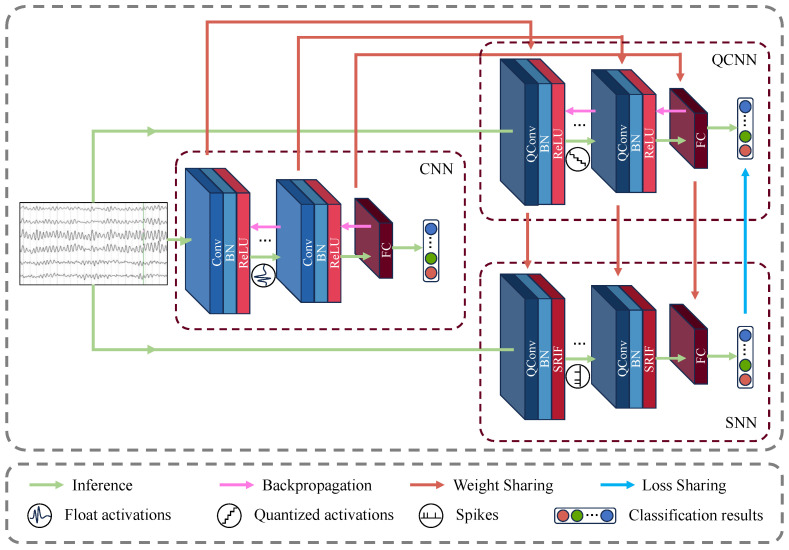
CT training framework. During training, the quantized network and the SNN share identical weights. By monitoring the classification accuracy of the SNN, the quantized network continuously applies targeted adjustments to the shared parameters to some extent to improve the performance of SNN.

In previous studies, researchers often mentioned that the disadvantage of the conversion method of ANNs to SNNs is that the SNN obtained by the conversion method is only an imitation of an ANN, which is mainly because the SNN did not participate in any training process. We have noticed that some methods try to share the weight of a SNN into an ANN [29,30,31], so that the ANN can pay attention to the performance of the SNN, and thus get a better SNN, which provides ideas for our research. We propose a CT training, where the core idea is to train the ANN and SNN together. Specifically, we use the classification results of the SNN to calculate the cross entropy loss. It expresses the SNN in the process of inference and the ANN in the process of backpropagation. The basis of this training method depends on the real-time weight sharing in the training process and the accurate representation of the ANN activation value. The specific process is shown in Figure 3.

The training process is divided into three stages. The first stage is the pre-training stage. By training a basic ANN, the weight value of the model foundation is obtained. The second stage is the clipping stage. Through the clipping method, the pre-trained ANN weights and activation values are quantified to provide a simple form for spike representation. At the same time, the existence of the quantized network ensures that the weight values can still maintain the excellent performance of the model in the discrete form. The third stage is the joint fine-tuning stage. In this stage, we use the weight sharing method to make the quantitative ANN supervise the SNN training situation in real time, so as to perform further fine-tuning. It is important to note that the proposed method becomes ineffective when the discrepancy between ANN and SNN is substantial. Therefore, the premise of successfully performing SNN fine tuning in CT training is that spiking neurons can accurately represent the activation value of ANN. Moreover, the training approach outlined here furnishes only a trim framework for exploiting the intrinsic properties of SNNs and, by itself, is insufficient to train a SNN from scratch. Figure 3 shows the entire training process. In fact, this method can support other ANNs with similar architectures, such as EEGNet and ShallowConvNet. In the experimental stage, we use these two networks for verification.

The superior time feature extraction ability of SNNs is more suitable for the decoding task of EEG signals. However, directly converting ANNs to SNNs does not improve performance, so CT training is essential to enhance the SNN’s learning capability. Overall, our method discretizes the weights and activation values of a pre-trained ANN network using trim techniques, allowing SRIF neurons to easily achieve fine-grained representations.

## 4. Experiments and Results

### 4.1. Experimental Setup

In this study, we evaluated our method on two MI datasets, BCI IV-2a and IV-2b [32], in order to visually and fairly demonstrate the effectiveness of our approach. The BCI IV-2a dataset contains EEG signals from nine participants who participated in four different MI tasks: imagining movements of the left hand, right hand, feet, and tongue. Each participant participated in the experiment within two days, conducting 288 trials per day and 72 trials per MI category. The BCI IV-2b dataset contains EEG signals from nine participants who participated in two different MI tasks: imagining the left and right hands. Each participant participated in feedback and no feedback experiments, with 240 trials performed in the feedback paradigm and 480 trials performed in the no feedback paradigm.

During preprocessing, we applied bandpass filtering between 4–40 Hz to EEG signals. This preprocessing process was performed on two datasets, BCI IV-2a and IV-2b. We used a window of 1.5–6 s for 2a, and 2.5–7 s for 2b [33]. For each subject, 80% trials were randomly selected by a stratified sampling method for training, and the remaining trials were used for testing.

We evaluated two established classification architectures in the domain of EEG signal analysis to validate the effectiveness of our method for our analysis, namely EEGNet [34] and ShallowConvNet [35]. EEGNet is a compact EEGNet signal analysis architecture that effectively captures differentiated EEG information and performs well in different BCI tasks. It operates on two dimensions of EEG signals (time and channel) and integrates the extracted features. ShallowConvNet is a convolutional neural network used for decoding EEG signals, specifically designed to decode frequency band power features in EEG signals. It has two convolutional layers and one pooling layer, where the first convolutional layer extracts temporal features and the second convolutional layer used to extract spatial features.

It should be noted that in order to meet the requirement for converting ANNs to SNNs, we are using the version of ShallowConvNet with the ReLU activation function so that the average spike firing rate of the IF neuron can be equivalent to the ANN activation value.

The proposed method was implemented using PyTorch (Version 2.2.0). All experiments were conducted on an NVIDIA RTX 4060 GPU (NVIDIA Corporation, Santa Clara, CA, USA). For SNN parameters, we set the number of time steps T=4. We employed the NAdam optimizer for loss function optimization for the model parameters, with an initial learning rate of 0.01 that was reduced by a factor of 10 after 150 epochs. In the initial training process of the ANN, we trained a total of 250 epochs, and in the subsequent CT training process, the number of epochs was set to 80.

### 4.2. ANNs to SNNs Analysis

MI is an important task type in the analysis of sequence signals, and our evaluation focuses on the decoding performance of two networks on two MI datasets. Table 1 shows the performance of our method using two classification models on two MI datasets.

**Figure 4:**
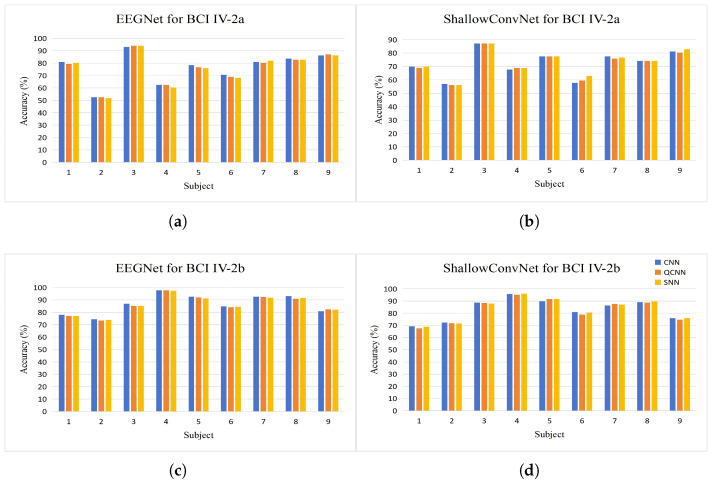
Performance comparison of defferent models on different datasets. (**a**) EEGNet for BCI IV-2a. (**b**) ShallowConvNet for BCI IV-2a. (**c**) EEGNet for BCI IV-2b. (**d**) ShallowConvNet for BCI IV-2b.

According to Table 1, the decoding accuracies of the converted SNNs are highly consistent with those of the original CNNs across both MI datasets. When using EEGNet, the performance of the SNN lagged behind that of the CNN by the maximum extent of 0.91% on average; while when using ShallowConvNet, the average performance of the SNN led that of the CNN by 0.69%. For most experimental settings, paired *t*-tests showed no statistically significant difference between SNN and CNN performance (*p* > 0.05). Only in the EEGNet (2b) configuration did the t-test indicate a significant difference (*p* = 0.0468), though the absolute accuracy gap was small. Furthermore, the two one-sided tests (TOST) [36] procedure with a predetermined equivalence margin of 2% (Δ=0.02) was conducted to assess the equivalence of the classification performance. All *p*-values yielded from the TOST procedure under four configurations are below 0.05, providing statistical evidence that the classification performances of the SNN and CNN are equivalent within the specified margin, which verifies the effectiveness of our method in reducing errors during the transfer process. To further observe the specific performance of our method, Figure 4 presents the results for each subject.

Based on the experimental results of Figure 4, the difference between the CNN, QCNN, and SNN is very small, and the largest error comes from the EEGNet model on the 8th subject of the BCI IV-2b dataset. At this time, the CNN achieved 93.04% accuracy versus 91.74% for the SNN. Notably, SNN performance occasionally exceeds CNN performance, for example, when the ShallowConvNet model was used on subject 6 of the BCI IV-2a dataset, the accuracy of the CNN was 57.76%, and the accuracy of the SNN was 62.93%. This performance improvement stems from the collaborative fine-tuning process, which is difficult to achieve with traditional transformation methods.

### 4.3. Ablation Experiments

To separately validate the effectiveness of the SRIF neuron and the CT strategy, we compared the performance of our proposed framework Q_CT_SRIF with that of three variants, all based on quantized networks. These variants include one using only IF neurons (Q_IF), one using only SRIF neurons (Q_SRIF), and one combining IF neurons with the CT strategy (Q_CT_IF). The results show that performance degrades when either the SRIF neuron or the CT training framework is removed from the full framework. To further validate the performance of Q_CT_SRIF, we added another configuration experiment C_IF, in which the trained ANN weights are transferred directly to SNNs with IF neurons without introducing the quantized network.

Table 2 presents the differences between each method and Q_CT_SRIF. The Q_CT_SRIF framework achieves higher mean accuracy than all other evaluated variants across all four settings, and most intermediate methods exhibit statistically significant differences from the proposed Q_CT_SRIF framework. Meanwhile, we noticed that both the SRIF neuron and the CT strategy contribute to performance gains, and compared to the performance improvement brought by CT training methods, the average performance improvement brought by SRIF is slightly higher. In addition, it can be clearly seen from Table 2 that the conversion method without introducing a quantized network has inferior performance, especially in the BCI IV-2a dataset.

### 4.4. Sensitivity Analysis of the Number of Time Steps *T*

**Figure 5:**
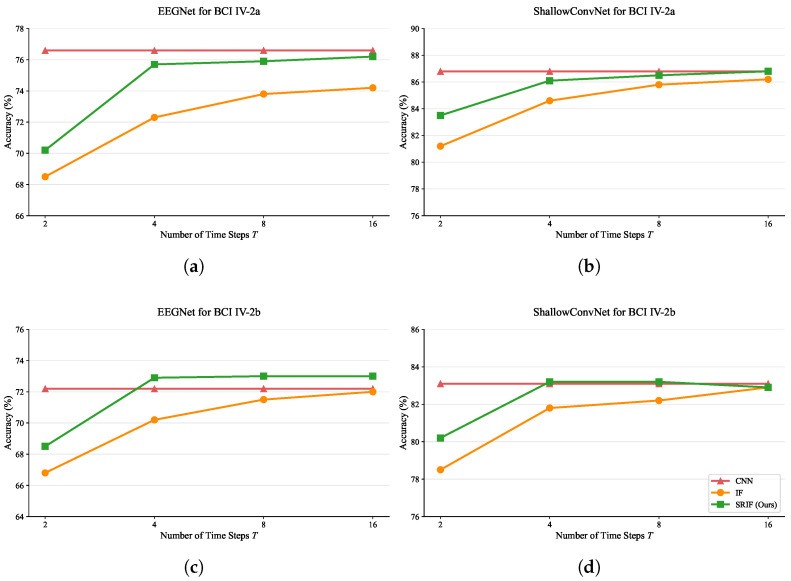
Accuracy for various numbers of time steps *T*. (**a**) EEGNet for BCI IV-2a. (**b**) ShallowConvNet for BCI IV-2a. (**c**) EEGNet for BCI IV-2b. (**d**) ShallowConvNet for BCI IV-2b.

The number of time steps *T* is a critical hyperparameter in SNN-based decoding, as it directly influences the trade-off between temporal resolution, computational latency, and classification performance. To rigorously justify our choice of T=4 and evaluate the robustness of the proposed SRIF method, we conducted a sensitivity analysis over different values of *T*, as shown in Figure 5.

It can be seen that for spiking models, both the IF and SRIF method exhibit a clear trend of improved accuracy as *T* increases, which can be attributed to richer temporal feature extraction and reduced asynchronous errors with more time steps. However, when T≥4, the rate of performance gain decelerates at larger *T*, while computational latency increases linearly with the number of time steps. Considering the balance between accuracy, delay and computational cost, we finally choose T=4.

### 4.5. Computational Efficiency

In order to verify the low energy consumption characteristics of SNNs, we conducted corresponding energy consumption analysis, which is an important method for evaluating the inference cost of a model. Multiply–accumulate (MAC) operations are a commonly used metric for CNNs, while for SNNs, we utilize accumulate (AC) operations. Specifically, the operational count of a SNN model is defined as [37]:

(12) $$AC=\sum_{l=1}^{n}\sum_{t=1}^{T}s_{l}\left(t\right)+\sum p_{in},$$

where *n* represents the number of model layers, and $\sum p_{in}$ represents the total number of input data. The results are presented in Table 3.

From Table 3, it can be observed that the SNN with the SRIF neuron exhibits a slight increase in computational operations compared to the SNN with the IF neuron. Notably, convolutional neural networks are quantified using MAC operations, whereas spiking neural networks rely on event-driven AC operations. As demonstrated in prior work [37], one MAC operation incurs higher computational overhead than a single AC operation. Even with the slight increment introduced by SRIF, the overall operational count of our SNN remains far lower than that of the corresponding CNN. These results confirm that the proposed method can maintain the inherent computational efficiency of SNNs.

### 4.6. Comparison with Other Methods

To further validate the superiority of the proposed approach, we benchmark it against two categories of state-of-the-art techniques:

- SNNs trained with the gradient-replacement paradigm, hereafter referred to as direct-training, and
- ANN-to-SNN conversion strategies specifically designed for EEG signals, denoted as ANN-to-SNN conversion methods.

From Table 4, it can be seen that our method produces SNNs with low conversion errors and low time step requirements. For example, in BCI IV-2a, compared to SCNet [31] using adaptive encoding and HR-SNN [38] using Ben’s Spike Algorithm (BSA) encoding, our method utilizes the first layer convolution for automatic spike encoding under the function of SRIF and a quantized network, which reduces *T*. Meanwhile, compared to LENet trained using surrogate gradients, although our method’s EEGNet and Shallow ConvNet did not surpass LENet in terms of final classification ability, our method can reduce the *T* requirements, and our method can be quickly applied to various ANNs compared to LENet to transform them into well-trained SNNs. While being lighter, our final performance did not significantly lag behind, and on BCI IV-2b, our method achieved lower *T* and parameter counts, while performing better than LENet [11]. It should be noted that the upper limit of the SNN obtained by our method is not limited to this, and a better ANN model will further enhance the performance of the SNN trained by our method. In this process, our focus is on achieving low-error and high-performance ANN to SNN conversion, converting well-trained ANNs in the task of decoding EEG signals into SNNs.

## 5. Discussion

Ablation experiments comparing the SRIF neuron model with the CT training method reveal that both components contribute to the performance of the proposed framework. However, it should be noted that the effectiveness of the two will also vary in different networks. For example, when the network layers are shallow, there is not much chance for the accumulated asynchronism error to manifest layer by layer. Therefore, neurons that can accurately represent ANN activation values are crucial, and the results in Table 2 can also verify this. Our experimental validation focused on shallow network architectures, which is also related to the characteristics of EEG signals themselves. In MI tasks, shallow networks can effectively accomplish classification [34,35]. Moreover, in contrast to image data, EEG signals exhibit intricate spectral compositions, and non-linear and non-stationary dynamics. Consequently, the precise representation of activation values becomes paramount, particularly in the initial layer of an SNN, which serves as an encoder transforming raw EEG waveforms into spike trains, enabling subsequent network layers accurately extract preliminary EEG features and to achieve accurate classification.

In this study, SRIF spiking neurons enhance representation precision by extending the activation values’ representation range through a two-stage spiking mechanism. At the same time, rationally determining the spike thresholds is also crucial for improving representation precision. However, it remains challenging to obtain optimal thresholds during SNN training. In IF networks, BN layers are typically introduced to normalize activations, and in such cases, the threshold is often simply set to 1. Alternatively, the threshold can be set to the maximum activation value or a certain percentile thereof [9]. These approaches often result in performance degradation due to inconsistencies between the resulting activation values and the distribution of ANN activations. The SRIF neurons we propose can effectively adapt to these straightforward threshold settings, and the rectification stage of spike calculation offers further opportunities for improvement. For instance, by introducing adaptive thresholds to refine the granularity of activation representation, we anticipate a significant reduction in such errors, thereby achieving a more accurate characterization of EEG signals [34].

**Figure 6:**
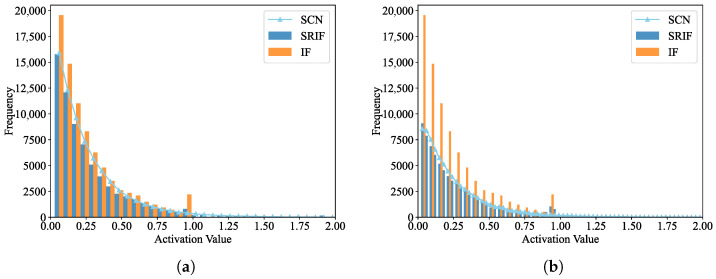
Representation capabilities of IF and SRIF neurons for ShallowConvNet (SCN) activation values. (**a**) Histogram of activation values when the firing thresholds of both IF and SRIF neurons are set to 1. (**b**) Histogram of activation values when the firing threshold of IF neurons is 1 and that of SRIF neurons is 0.5.

This phenomenon is illustrated in Figure 6. In Figure 6a, both the SRIF and IF spike thresholds are set to 1. It can be observed that the activation value distribution represented by SRIF is not limited to within the spike threshold, and more closely resembles the activation value distribution of an ANN. In contrast, the activation value range represented by IF neurons is constrained within the threshold, and significant clipping errors result in a considerable deviation from the activation value distribution of the ANN. Furthermore, Figure 6b demonstrates the finer representational capability of SRIF. When the spike threshold of SRIF is set to half that of IF, IF can only represent a single spiking activation value within an activation interval, whereas SRIF is able to represent two activation values that more accurately reflect the true activation value.

In this study, the objective of our spiking neuron component was to devise a unit whose spike dynamics accurately encode the instantaneous activation of the associated layer. Indeed, our study concentrates on the local layers of the overall network, thereby lacking a macroscopic perspective. Although we initially attempted to address this limitation through CT training, more sophisticated perceptual mechanisms are necessary to establish a bidirectional, information-theoretic alignment between ANN and SNN representations. Specifically, by implementing information sharing related to activation values, the training effect that can be achieved should be more significant. For instance, a layer-wise similarity analysis that quantifies the discrepancy between the ANN and SNN enables the ANN to dynamically calibrate its training trajectory in real time. Similarity analysis reveals that solely relying on loss function design may inadequately address feature discrepancies. Concurrently, we observe that even with a more judiciously designed loss, the CT training still exhibits non-negligible headroom for improvement. In our framework, for instance, spike encoding is delegated to the first convolutional layer; the resulting encoding error propagates and markedly compromises the eventual network performance. Consequently, explicitly penalizing the mismatch between the encoding layers of the QCNN and the target SNN within the loss formulation is expected to further enhance the latter’s discriminative capability. Extending this idea, layer-wise supervision that minimizes the activation discrepancies between the two networks at every depth could potentially alleviate the well-documented degradation of deep SNNs. However, the crux lies in devising an effective weighting strategy for aggregating the per-layer losses, a challenge that will constitute the primary focus of our forthcoming study.

This study verifies the feasibility and effectiveness of the proposed SRIF neuron and CT training framework in EEG-based MI classification. It achieves classification performance comparable to that of ANNs while retaining the superior energy efficiency and lower computational complexity of SNNs. However, the experiments on two widely used datasets are subject-dependent. For real-time BCI applications, the generalization capability of the algorithm across subjects, datasets, and complex noisy environments is crucial. In future work, we will further investigate improvements to the conversion framework by integrating advanced ANN architectures and conduct validations from software experiments to practical hardware deployment.

## 6. Conclusions

In this study, we propose a self-rectifying integrate-and-fire neuron and CT training method, and achieve the construction of a SNN with higher accuracy and lower quantization error and asynchronism error, while achieving effective MI classification tasks. We propose rectification spikes in SRIF to balance the excessive fire of spikes that cause clipping error and asynchronism error. Furthermore, we introduce a quantized ANN that jointly discretizes both activations and weights, thereby significantly reducing the clipping error incurred when the SNN encodes ANN activations via average firing rates. This clipping scheme is embedded into the CT framework, enabling synergistic optimization of the quantized network and the SNN through weight sharing. Finally, we have implemented the conversion of commonly used EEG signal decoding networks EEGNet and ShallowConvNet into the SNN, achieving effective EEG decoding.

In future studies, we will focus on improving the spike firing mechanism modeling of SRIF to reduce conversion errors, while also exploring effective domain adaptation training frameworks to enhance the generalization performance of the SNNs across subjects and datasets, thereby further promoting the development of SNNs in the field of BCI.

## Figures and Tables

**Table 1:** Performance comparison between original CNNs and SNNs on the BCI IV-2a (2a) and IV-2b (2b) datasets.

	EEGNet (2a)	EEGNet (2b)	ShallowConvNet (2a)	ShallowConvNet (2b)
CNN	**76.58 ± 11.87**	**86.79 ± 7.45** *	72.18 ± 9.6	83.1 ± 8.48
SNN	75.67 ±12.55 ^†^	86.1 ± 7.2 ^†^	**72.87 ± 9.11** ^†^	**83.2 ± 8.93** ^†^

Bold font is used to indicate the best outcome. “*” represents significant differences (*p* < 0.05, paired *t*-test) compared to SNN. “^†^” represents the equivalence confirmed by TOST procedure (*p* < 0.05, equivalence margin Δ=0.02) between CNN and SNN.

**Table 2:** Performance comparison across different models and methods.

	EEGNet (2a)	EEGNet (2b)	ShallowConvNet (2a)	ShallowConvNet (2b)
C_IF	58.06 ± 11.28 *	83.19 ± 9.14 *	51.46 ± 8.56 *	80.35 ± 8.32 *
Q_IF	72.3 ± 13.99 *	84.57 ± 7.5 *	70.18 ± 9.65 *	81.77 ± 9.44 *
Q_SRIF	73.99 ± 12.5 *	86.02 ± 7.25	71.91 ± 10.21	82.46 ± 9.01 *
Q_CT_IF	74.18 ± 13.43 *	84.98 ± 7.37 *	71.16 ± 9.46 *	82.17 ± 9.46 *
Q_CT_SRIF	**75.68 ± 12.55**	**86.1 ± 7.2**	**72.87 ± 9.11**	**83.2 ± 8.93**

Bold font is used to indicate the best outcome. “*” represents significant differences (*p* < 0.05, paired *t*-test) compared to Ours.

**Table 3:** Computational efficiency comparison of CNN and SNN models. CNNs are measured in MAC operations, while SNNs are measured in AC operations.

Model	MAC/AC FLOPs
EEGNet (CNN)	1.98 × 10^6^ MAC FLOPs
EEGNet (SNN with IF)	**6.14 × 10^4^** AC FLOPs
EEGNet (SNN with SRIF)	8.14 × 10^4^ AC FLOPs
ShallowConvNet (CNN)	2.79 × 10^7^ MAC FLOPs
ShallowConvNet (SNN with IF)	**3.02 × 10^6^** AC FLOPs
ShallowConvNet (SNN with SRIF)	5.33 × 10^6^ AC FLOPs

Bold font is used to indicate the best outcome.

**Table 4:** Performance comparison with existing advanced methods.

Dataset	Model Type	Train Type	Models	Accuracy	*T*	Parameters
BCI IV-2a	ANN	-	1D-CNN [39]	72.59	-	206,484
			BrainGridNet [40]	75.28	-	16,700
			EEG-Inception [41]	80.42	-	8,396,932
			ShallowConvNet [35]	72.18	-	**2844**
			EEGNet [34]	76.58	-	4784
			CRGNet [42]	**82.07**	-	486,874
	SNN	Direct-training	NeuCube [10]	52.88	-	>500,000
			SCNN [43]	73.48	250	272,452
			LENet (Max) [11]	**77.84**	**100**	**3264**
		ANNs to SNNs	HR-SNN [38]	**77.58**	1000	4570
			SCNet [31]	70.42	10	**2632**
			ShallowConvNet-CT-SRIF (this work)	72.87	**4**	2844
			EEGNet-CT-SRIF (this work)	75.67	**4**	4784
BCI IV-2b	ANN	-	LENet [11]	78.95	-	2320
			ShallowConvNet [35]	83.1	-	**2084**
			EEGNet [34]	**86.79**	-	4214
	SNN	Direct-training	LENet (Max) [11]	79.17	100	2320
		ANNs to SNNs	SCNet [31]	85.18	10	2632
			ShallowConvNet-CT-SRIF (this work)	83.2	**4**	**2084**
			EEGNet-CT-SRIF (this work)	**86.1**	**4**	4214

Bold font is used to indicate the best outcome.

## Data Availability

The two datasets used in this study, namely BCI Competition IV Dataset 2a and BCI Competition IV Dataset 2b, are both publicly available. The dataset link is as follows: https://www.bbci.de/competition/iv/ (accessed on 2 April 2026). The original codes presented in this study are openly available at https://github.com/hdumm/SRIF-CT-SNN (accessed on 12 May 2026).
